# NHANES-based analysis of the correlation between leisure-time physical activity, serum cotinine levels and periodontitis risk

**DOI:** 10.1186/s12903-024-04141-9

**Published:** 2024-04-17

**Authors:** Hua Fu, Diya Zhang, Yining Li

**Affiliations:** 1 Shangyu people’s Hospital, Shaoxing City, Zhejiang Province 312300 China; 2 Run Run Shaw Hospital, Zhejiang University, Hangzhou City, Zhejiang Province China; 3 Zhejiang University Affiliated Stomatology Hospital, Hangzhou City, Zhejiang Province China

**Keywords:** Leisure time physical activity, Smoking, Cotinine, Periodontitis

## Abstract

**Objective:**

To investigate the association of leisure-time physical activity and serum cotinine levels with the risk of periodontitis in the general population and to further analyze the interaction between leisure-time physical activity and serum cotinine levels on the risk of periodontitis.

**Methods:**

This was a cross-sectional study, extracting data from 9605 (56.19%) participants in the National Health and Nutrition Examination Survey (NHANES) database from 2009 to 2014, and analyzing the relationship and interaction effects of serum cotinine level, leisure time physical activity, and risk of periodontitis by weighted univariate logistic modeling; Effect sizes were determined using ratio of ratios (OR), 95% confidence intervals (95% CI).

**Results:**

5,397 (56.19%) of 9,605 participants had periodontitis; an increased risk of periodontitis was found in those in the leisure time physical activity intensity < 750 MET × min/week group (OR = 1.44, 95% CI: 1.17–1.78). Serum cotinine levels ≥ 0.05 ng/ml were associated with an increased risk of periodontitis (OR = 1.99, 95% CI: 1.69–2.33). The group with low leisure physical activity and serum cotinine levels ≥ 0.05 ng/ml had an increased risk of periodontitis compared to the group with high leisure physical activity and serum cotinine levels < 0.05 ng/ml (OR = 2.48, 95% CI: 1.88–3.27). Interaction metrics RERI = 0.90 (95% CI: 0.44–1.36) and API = 0.36 (95% CI: 0.18–0.55); CI for SI = 2.55 (95% CI: 1.03–6.28). for API 0.36.

**Conclusion:**

Leisure time physical activity intensity interacted with smoking exposure on periodontitis risk and may provide the general population with the opportunity to Increasing leisure-time physical activity and smoking cessation may provide recommendations for the general population.

## Preamble

Periodontitis is one of the common periodontal diseases, which is mainly a chronic inflammation of periodontal support tissues caused by localized factors, with onset more common after the age of 35 years, affecting 70-80% of adults, and is a major cause of tooth loss in adults [1] Data from the 2009–2014 American Health and Nutrition Examination Survey (AHANES) show [2] that 42% of U.S. adults suffer from periodontitis, with 7.8% of them have severe periodontitis, which is associated with loss of periodontal support with a significant decline in chewing function and tooth loss, and may have a significant negative impact on oral health-related quality of life. In addition to its impact on oral health, periodontitis is associated with the risk of chronic diseases such as cardiovascular disease (CVD) and Alzheimer’s disease [3]. The socio-economic impact of periodontitis in the United States and Europe was estimated to be approximately $154 billion and €159 billion in 2018 [4]. The impact of periodontitis on patients’ quality of life and overall health has been widely validated in recent decades [5]. Therefore, it is important to identify modifiable influencing factors to prevent and control the onset and progression of periodontitis and to reduce the disease burden. Adverse lifestyle habits are important modifiable factors that influence chronic diseases such as periodontitis [6]. There is evidence that tobacco exposure promotes the development and progression of periodontitis by impairing immune and vascular mechanisms, with smokers having a significantly higher prevalence of periodontitis than non-smokers [7]; among non-smokers, high levels of tobacco exposure are also associated with an increased risk of periodontitis [8]. Nicotine, a metabolite of nicotine, is considered a key biomarker of tobacco smoke exposure [9]. The health benefits of moderate physical activity have received much attention in recent years. A systematic review confirmed [10] that leisure time physical activity is a potential tool to reduce the prevalence of periodontal disease. Leisure time physical activity is defined as physical exercise performed during free time and not required as part of the basic activities of daily living. It is performed at the discretion of the subject and includes activities such as sports, exercise and leisure walks. Leisure time physical activity is recognized as a preventive factor for most chronic non-communicable diseases [11]. In fact, dynamic exercise and mental health associated with leisure time physical activity is associated with improved cardiovascular metabolic function and reduced low-grade systemic inflammation [12, 13]. In contrast, subjects who were less physically active in their leisure time had higher rates of cardiovascular disease, type 2 diabetes, cancer, depression, and generally shorter life expectancy [14]. Results of a study conducted in a US population suggest that high leisure time physical activity is associated with a reduced risk of periodontitis [15]. Previously, Correia et al. [16] found that cigarette smoke exposure and physical training interacted on the inflammatory and oxidative profiles of mouse muscle. Therefore, we hypothesized that high levels of leisure-time physical activity may be associated with a reduced risk of periodontitis, whereas tobacco exposure may increase the risk of periodontitis, and that there may be an interaction between the level of leisure-time physical activity and the level of tobacco exposure on the risk of periodontitis.

This study will use data from the National Health and Nutrition Examination Survey (NHANES) to explore the correlation between leisure time physical activity and serum cotinine levels (although urinary cotinine levels are more stable than serum cotinine levels, no data related to urinary cotinine were seen in the NHANES, so serum cotinine will be used in this study) and the risk of periodontitis in the general population, and to analyze the interactive effect of leisure time physical activity and serum cotinine levels on periodontitis risk.

## Research methodology

### Study design and population

The population data used in this study were extracted from the National Health and Nutrition Examination Survey (NHANES).The NHANES database is collected from a nationally representative sample of U.S. civilians using a stratified multistage sampling design.The purpose of the NHANES database is to monitor the nutritional and health status of adults, and children across the U.S [17]. Details of the survey design and methodology can be found on the NHANES Web site [Centers for Disease Control andPrevention (CDC), http://cdc.gov/nchs/nhanes]. Ethical approval for all NHANES cycles was obtained from the National Center for Health Statistics (NCH) of the Centers for Disease Control and Prevention (CDC). The National Center for Health Statistics (NCHS) Research Ethics Review Board (ERB) approved and all survey participants gave written informed consent. The population from the NHANES database for six consecutive years, 2009–2010, 2011–2012, and 2013–2014, was combined for this paper. For this study, records were extracted from NHANES for investigators who met the following criteria: (1) had at least two teeth (excluding the third molar); (2) had a periodontal examination; (3) had complete information on physical activity; and (4) had received a serum cotinine test from NHANES 2009–2014. Participants for whom there were no significant covariates were excluded.

**Fig. 1:**
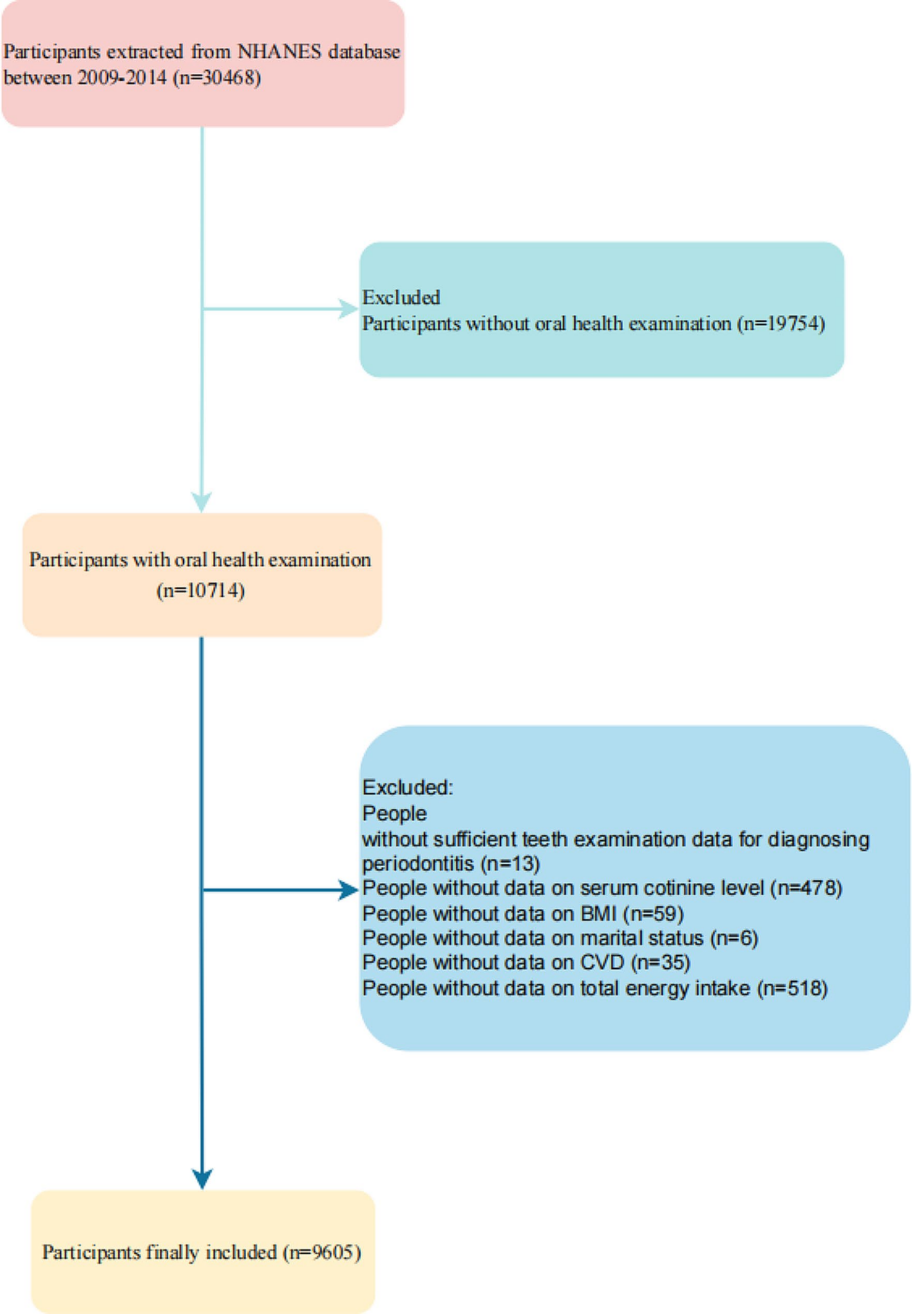
Flowchart for screening the study population

A total of 30,468 participants were identified. Excluding 19,754 participants who did not have an oral health checkup, to further exclude 13 who did not have sufficient dental examination data for the diagnosis of periodontitis, continue to exclude 478 who did not have serum cotinine levels, again exclude 59 who did not have data on body mass index, 6 who did not have marital status, and 35 with co-morbid cardiovascular disease, and finally exclude 518 who did not have data on total energy intake, for a total of 9605 participants (with a combined number of 43,260 teeth) were included in this study, and the detailed screening process is shown in Fig. 1.

### Potential covariates

Age (years), sex (female or male), race (Mexican American, non-Hispanic black, non-Hispanic white, other Hispanic, or other race, including multiracial), marital status (married or unmarried), poverty-to-income ratio (PIR) (0-1.13, 1. 13 − 5, or unknown), alcohol use (never, < 1 time/week, or ≥ 1 time/week), occupational sports activity, screen time (< 4 h, ≥ 4 h, or unknown), type 2 diabetes mellitus (yes or no), hypertension (yes or no), dyslipidemia (yes or no), cardiovascular disease (yes or no), nonsteroidal anti-inflammatory drugs (yes or no), non-steroidal anti-inflammatory drugs (yes or no), anti-infective drugs (yes or no), body mass index (BMI) (< 25 kg/m^2^ or ≥ 25 kg/m ^2^), vitamin D (VD) (< 50 nmol/L or ≥ 50 nmol/L), flossing/dental appliances (no, yes, unknown), total energy, and Mediterranean diet (MED) score were potential covariates.

### Main variables

Leisure time physical activity data were collected by a staff-administered questionnaire [18]. Leisure time physical activity was assessed on the basis of metabolic equivalent (MET) hours per week to take into account the intensity of activity and time spent [19]. Physical activity was reported in terms of the average number of hours spent per week over the past year. Physical activity levels are the product of the time spent on each activity multiplied by a specific MET value based on the Physical Activity Compendium. Leisure time physical activities included martial arts, gymnastics, dance, acrobatics, jogging, swimming, playing soccer, basketball or tennis, and playing badminton, volleyball, or table tennis. In this study, high leisure time physical activity was defined as ≥ 750 MET × minutes/week.

Serum cotinine levels: the Centers for Disease Control and Prevention (CDC) used isotope dilution-high performance liquid chromatography/atmospheric pressure chemical ionization tandem mass spectrometry [20] for serum cotinine concentrations, and the detection limits (DLs) used by the CDC were 0.05 ng/ml from 2009 to 2014, and according to the determination of cotinine concentrations The results were categorized into ≥ 0.05 ng/ml and < 0.05 ng/ml groups. Details of sample collection and processing are reported elsewhere by the CDC [21].

### Outcome variables

The outcome was the occurrence of periodontitis. All survey participants who were at least 30 years of age, had at least one tooth (excluding the third molar) and did not meet any of the health exclusion criteria were eligible to undergo a full-mouth periodontal examination [22], which was performed by a trained dental hygienist (2009–2010 cycle) and a general dentist (2011–2012 cycle and 2013–2014 cycle) using a color-coded periodontal probe (PCP2. HuFriedy) was performed. Gingival recession and probing pocket depths were measured at six locations on each tooth (excluding the third molar), and then clinical attachment levels were calculated. Periodontal examiners were trained and calibrated prior to the survey and then regularly 2–3 times per year [23]. Only participants who had a complete periodontal examination were considered in this study. The AAP/CDC criteria were applied to determine periodontitis [24], categorizing its severity as mild, moderate, or severe, and the percentage of teeth with periodontitis was determined based on all teeth present.

### Additive interaction modeling

Relative Excess Risk of Interaction (RERI), Attributable Proportion of Interaction (API), and Synergy Index (SI) are indicators of additive interactions, where RERI stands for Relative Excess Risk, RERI = R11-R10-R01 + 1, which describes the difference between the sum of the joint effects of the two variables and the difference between the single effects. When the confidence interval (CI) is 0, it indicates the absence of an interaction.API represents the attribution ratio, API = RERI/R11, which describes the proportion of the total effect that is attributable to the interaction, ranging from − 1 to + 1. When the CI is 0, there is no interaction.SI represents the interaction index, SI=(R11-1)((R10-1)×(R01-1)). When the CI confidence interval is 1, there is no interaction [25].

### Statistical analysis

The study used R software (R version 4.1.2) for all statistical analyses. Count data were expressed as number of cases and percentage [n (%)], and comparisons between groups were made using the chi-square test or Fisher’s exact probability method. Measurement data were expressed as mean and standard error (S.E.), and comparisons between groups were made using t-tests.NHANES makes the data collected nationally representative through sophisticated sampling designs and the use of sample weights. In this paper, we weighted the data according to the sample weight calculation method recommended by NHANES, combining the data for 6 years from 2009 to 2014, with the 6-year weight equal to 1/3 of the 2-year weight; the associations of serum cotinine levels and spare-time physical activity with the risk of periodontitis were analyzed with weighted logistic regression models, and the results were reported as the ratio of ratios (OR) and their 95% confidence intervals (95% CI) and p-values; no covariates were adjusted for in model 1, and age, gender, race, marital status, PIR, screen time, diabetes mellitus, hypertension, dyslipidemia, cardiovascular disease, NSAIDs, VD, flossing/dental appliances, total energy, and MED scores were adjusted for in model 2 to explore whether the associations differed across gender and age groups, with a two-sided p-value < 0.05 was considered statistically significant difference.

## Findings

### Comparison of the characteristics of the periodontitis group and the non-periodontitis group

The mean age was higher in the periodontitis group than in the non-periodontitis group (54.23 vs. 47.63 years). Serum cotinine levels ≥ 0.05 ng/mL were more frequent in the periodontitis group than in the non-periodontitis group (47.76% vs. 29.73%). The proportion of participants with leisure time physical activity intensity < 750 MET × min/week was higher in the periodontitis group than in the non-periodontitis group (89.53% vs. 81.13%). The proportion of study participants with comorbid diabetes mellitus was higher in the periodontitis group than in the non-periodontitis group (17.81% vs. 9.19%). More detailed information on the characteristics of periodontitis and non-periodontitis participants is presented in Table 1.

**Table 1:** Baseline characteristics of participants

Variant	Total (n = 9605)	No periodontitis (n = 4208)	Periodontitis (n = 5397)	Statistical information	*P*
Age, years, mean (S.E.)	50.80 (0.24)	47.63 (0.30)	54.23 (0.32)	*t*=-15.15	< 0.001
Gender, n (%)				*χ*^2^ = 149.215	< 0.001
females	4845 (50.69)	2571 (58.50)	2274 (42.25)		
male	4760 (49.31)	1637 (41.50)	3123 (57.75)		
Race, n (%)				*χ*^2^ = 116.957	< 0.001
Mexican American	1393 (7.96)	436 (5.54)	957 (10.57)		
Non-Hispanic blacks	1928 (10.16)	688 (7.84)	1240 (12.66)		
Non-Hispanic whites	4285 (69.86)	2183 (75.90)	2102 (63.33)		
Other Hispanics	951 (5.26)	397 (4.72)	554 (5.86)		
Other races - including multiracial	1048 (6.76)	504 (6.00)	544 (7.59)		
Marital status, n (%)				*χ*^2^ = 45.195	< 0.001
married	5630 (63.91)	2635 (68.94)	2995 (58.48)		
unmarried	3975 (36.09)	1573 (31.06)	2402 (41.52)		
PIR, n (%)				*χ*^2^ = 174.401	< 0.001
0-1.13	2608 (17.21)	858 (12.11)	1750 (22.73)		
1.13-5	6244 (76.43)	3063 (82.17)	3181 (70.24)		
uncharted	753 (6.35)	287 (5.72)	466 (7.04)		
Alcohol consumption, n (%)				*χ*^2^ = 5.451	0.066
Never drink	2390 (19.67)	1020 (19.19)	1370 (20.19)		
<1x/week	3525 (35.54)	1523 (34.20)	2002 (36.98)		
≥1 time/week	3690 (44.79)	1665 (46.61)	2025 (42.83)		
Serum cotinine levels, n (%)				*χ*^2^ = 203.503	< 0.001
<0.05 ng/ml	5583 (61.60)	2794 (70.27)	2789 (52.24)		
≥0.05 ng/ml	4022 (38.40)	1414 (29.73)	2608 (47.76)		
Physical activity in leisure time, n (percentage)				*χ*^2^ = 69.898	< 0.001
<750 m x minutes/week	8463 (85.17)	3526 (81.13)	4937 (89.53)		
≥750 Met x min/week	1142 (14.83)	682 (18.87)	460 (10.47)		
Occupational physical activity, meters x minutes/week, mean (S.E.)	447.21 (52.29)	369.89 (67.53)	530.80 (65.45)	*t*=-1.95	0.057
Screen usage time, n (%)				*χ*^2^ = 33.151	< 0.001
<4 h	3429 (38.30)	1727 (43.26)	1702 (32.94)		
≥4 h	2700 (29.41)	1210 (29.39)	1490 (29.43)		
uncharted	3476 (32.29)	1271 (27.35)	2205 (37.63)		
Diabetes, n (%)				*χ*^2^ = 125.921	< 0.001
hasn’t	7930 (86.67)	3714 (90.81)	4216 (82.19)		
be	1675 (13.33)	494 (9.19)	1181 (17.81)		
Hypertension, n (%)				*χ*^2^ = 73.439	< 0.001
hasn’t	4240 (47.59)	2204 (53.72)	2036 (40.96)		
be	5365 (52.41)	2004 (46.28)	3361 (59.04)		
Dyslipidemia, n (%)				*χ*^2^ = 18.837	< 0.001
hasn’t	2381 (24.78)	1195 (27.44)	1186 (21.91)		
be	7224 (75.22)	3013 (72.56)	4211 (78.09)		
Cardiovascular diseases, n (%)				*χ*^2^ = 192.089	< 0.001
hasn’t	8795 (92.88)	3999 (95.63)	4796 (89.90)		
be	810 (7.12)	209 (4.37)	601 (10.10)		
Non-steroidal anti-inflammatory drugs, n (%)				*χ*^2^ = 21.076	< 0.001
hasn’t	8397 (88.13)	3733 (89.52)	4664 (86.63)		
be	1208 (11.87)	475 (10.48)	733 (13.37)		
Anti-infective drugs, n (%)				*χ*^2^ = 0.220	0.639
hasn’t	9165 (94.87)	4001 (94.73)	5164 (95.02)		
be	440 (5.13)	207 (5.27)	233 (4.98)		
Body mass index, n (%)				*χ*^2^ = 3.582	0.058
<25 kg/m^2^	2520 (26.60)	1167 (27.60)	1353 (25.52)		
≥25 kg/m^2^	7085 (73.40)	3041 (72.40)	4044 (74.48)		
VD, n (%)				*χ*^2^ = 90.944	< 0.001
<50 mmol/L	2865 (22.38)	1064 (18.34)	1801 (26.75)		
≥50 nmol/L	6740 (77.62)	3144 (81.66)	3596 (73.25)		
Dental floss/instrumentation, n (%)				*χ*^2^ = 159.424	< 0.001
hasn’t	2998 (27.41)	1001 (22.07)	1997 (33.17)		
be	6533 (72.13)	3183 (77.49)	3350 (66.33)		
uncharted	74 (0.46)	24 (0.43)	50 (0.49)		
Total energy, kcal, mean (S.E)	2173.34 (14.98)	2149.40 (15.54)	2199.21 (22.32)	*t*=-2.11	0.040
MED score, mean (S.E.)	6.45 (0.05)	6.54 (0.06)	6.35 (0.05)	*t* = 3.14	0.003

S.E: standard error; PIR: poverty-to-income ratio; CVD: cardiovascular disease; BMI: body mass index: cardiovascular disease; BMI: body mass index; VD: MED: Mediterranean diet

### Relationship between leisure time exercise intensity or serum cotinine levels and risk of periodontitis

Age, gender, race, marital status, PIR, screen time, diabetes, hypertension, dyslipidemia, cardiovascular disease, nonsteroidal anti-inflammatory drugs, VD, flossing/dental appliances, total energy, and Med scores were covariates associated with risk of periodontitis. Individuals with a leisure-time physical activity intensity of < 750 MET × min/week may be associated with an elevated risk of periodontitis compared with those with a leisure-time physical activity intensity of ≥ 750 MET × min/week (OR = 1.99, 95% CI: 1.67–2.37). After adjusting for covariates, an increased risk of periodontitis was found among those in the leisure time physical activity intensity < 750 MET × min/week group (OR = 1.44, 95% CI: 1.17–1.78). In the adjusted model, serum cotinine levels ≥ 0.05 ng/ml were associated with an increased risk of periodontitis (OR = 1.99, 95% CI: 1.69–2.33) (Table 2).

**Table 2:** Relationship between intensity of amateur physical activity or serum cotinine levels and risk of periodontitis

Variant	Model 1		Model 2	
	OR (95% CI)	P	OR (95% CI)	P
Leisure sports activities				
≥750 MET x minutes/week	Ref		Ref	
<750 MET x minutes/week	1.99 (1.67–2.37)	< 0.001	1.44 (1.17–1.78)	< 0.001
Serum cotinine levels				
<0.05 ng/ml	Ref		Ref	
≥0.05 ng/ml	2.16 (1.88–2.48)	< 0.001	1.99 (1.69–2.33)	< 0.001

OR: prevalence; CI: confidence interval; Ref: reference value

Model 1: Unadjusted univariate logistic regression model

Model 2: Multivariate Logistic Regression Model Adjusting for Age, Gender, Race, Marital Status, PIR, Screen Time, Diabetes, Hypertension, Dyslipidemia, Cardiovascular Disease, NSAIDs, VD, Flossing/Dental Appliances, Total Energy, and MED Score

### Interaction between leisure time physical activity and serum cotinine levels on periodontitis risk

**Fig. 2:**
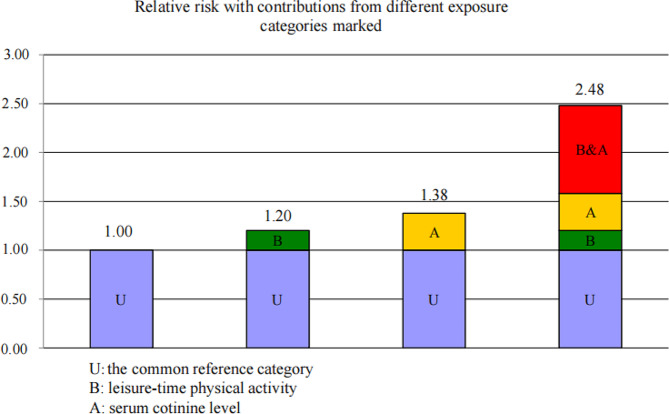
Relative risk contribution of amateur physical activity and serum cotinine levels to the development of periodontitis

Additive interaction terms for leisure time physical activity & serum cotinine levels included high leisure time physical activity & serum cotinine levels < 0.05 ng/mL, low leisure time physical activity & serum cotinine levels < 0.05 ng/mL, high leisure time physical activity & serum cotinine levels ≥ 0.05 ng/mL, and low leisure time physical activity & serum cotinine levels ≥ 0.05 ng/mL. after adjusting for confounders, the group with low leisure time physical activity & serum cotinine levels ≥ 0.05 ng/ml had an increased risk of periodontitis compared to the group with high leisure time physical activity & serum cotinine levels < 0.05 ng/ml (OR = 2.48, 95% CI: 1.88–3.27) (Table 3). The interaction metrics RERI = 0.90 (95% CI: 0.44–1.36) and API = 0.36 (95% CI: 0.18–0.55) had a CI excluding 0 and > 0; SI = 2.55 (95% CI: 1.03–6.28) had a CI excluding 1 and > 1. The API of 0.36 indicated that 36% of the investigators in our study had periodontitis attributed to low leisure time physical activity and serum cotinine levels ≥ 0.05 ng/mL (Table 3). Figure 2 shows the relative risk of leisure time physical activity and serum cotinine levels on the development of periodontitis. Sensitivity analyses showed that there was no significant difference between deleting or not deleting the missing values.

**Table 3:** Interaction between amateur physical activity and serum cotinine levels on the risk of periodontitis

Variant	Model 1		Model 2	
	OR (95% CI)	P	OR (95% CI)	P
Serum cotinine levels < 0.05 ng/ml & Lots of leisure time sports activities	Ref		Ref	
Serum cotinine level < 0.05ng/ml & Low leisure time physical activity	1.70 (1.38–2.09)	< 0.001	1.20 (0.94–1.53)	0.135
Serum cotinine level ≥ 0.05ng/ml & Lots of leisure time sports activities	1.70 (1.21–2.37)	0.003	1.38 (0.94–2.03)	0.101
Serum cotinine level ≥ 0.05 ng/ml & Low leisure time physical activity	3.66 (2.90–4.61)	< 0.001	2.48 (1.88–3.27)	< 0.001
RERI (95% CI)	1.29 (0.77–1.81)		0.90 (0.44–1.36)	
API (95% CI)	0.35 (0.22–0.48)		0.36 (0.18–0.55)	
SI (95% CI)	1.94 (1.34–2.80)		2.55 (1.03–6.28)	

OR: prevalence; CI: confidence interval; Ref: reference value; RERI: relative excess risk due to interaction; API: attributable proportion of interaction; SI: synergy index: attributable proportion of interaction; SI: synergy index

Model 1: Unadjusted univariate logistic regression model

Model 2: Multivariate Logistic Regression Model Adjusting for Age, Gender, Race, Marital Status, PIR, Screen Time, Diabetes, Hypertension, Dyslipidemia, Cardiovascular Disease, NSAIDs, VD, Flossing/Dental Appliances, Total Energy, and MED Score

### Subgroup analysis of the interaction between leisure time physical activity and serum cotinine levels on the risk of periodontitis

**Fig. 3:**
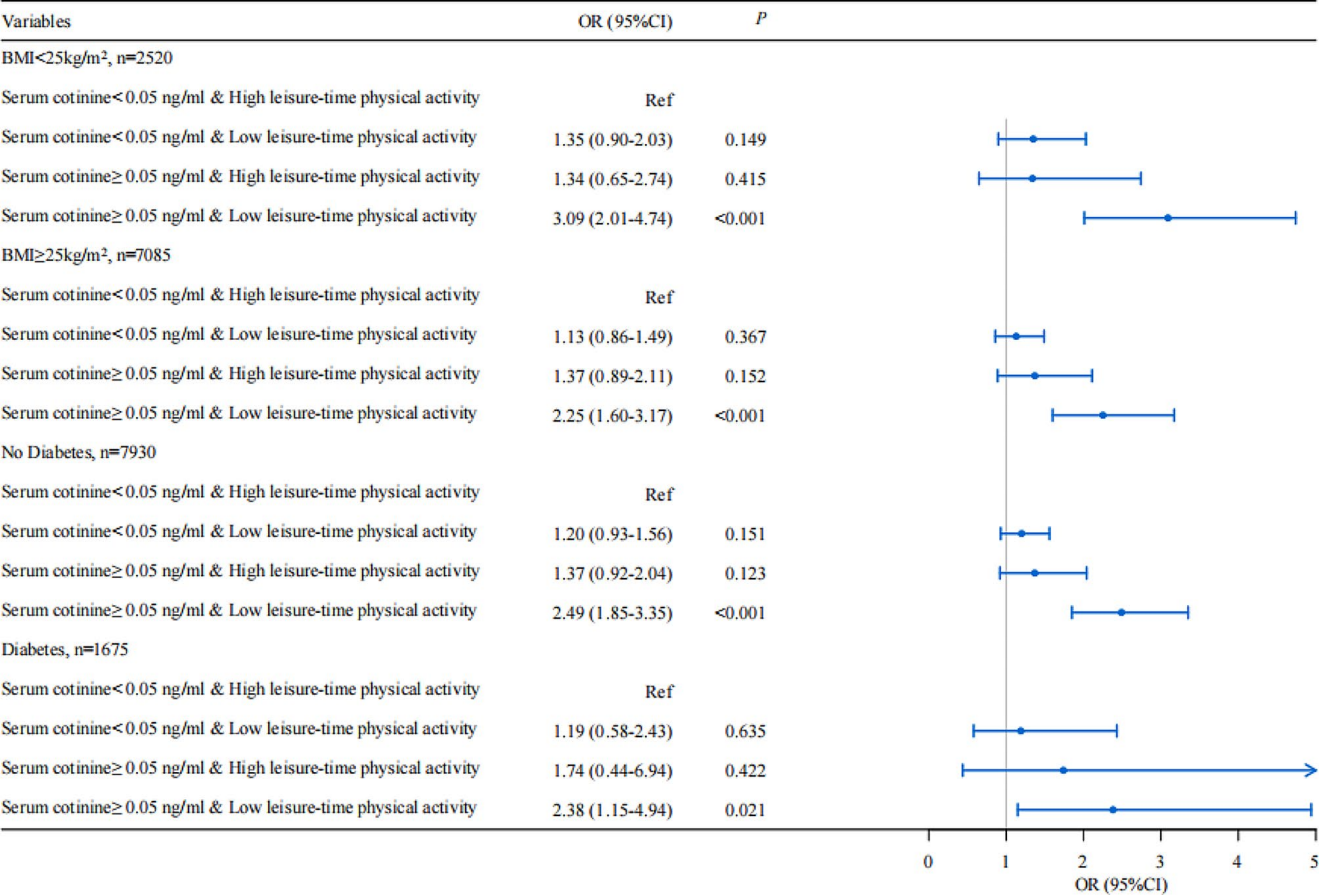
Subgroup analysis of the interaction between leisure time physical activity and serum cotinine levels on the risk of developing periodontitis

Among people with a body mass index < 25 kg/m^2^, those with serum cotinine levels ≥ 0.05 ng/mL and low leisure-time physical activity may have an increased risk of periodontitis compared with those with serum cotinine levels < 0.05 ng/mL and high leisure-time physical activity. Among those with a body mass index ≥ 25 kg/m^2^, those with serum cotinine levels ≥ 0.05 ng/mL and low leisure-time physical activity had a higher risk of periodontitis compared with those with serum cotinine levels < 0.05 ng/mL and high leisure-time physical activity. In both diabetic and non-diabetic groups, the risk of periodontitis was observed to be higher in those with serum cotinine levels ≥ 0.05 ng/mL and low spare-time physical activity than in those with serum cotinine levels < 0.05 ng/mL and high spare-time physical activity (Fig. 3).

## Discussion

Periodontitis is a chronic infectious disease with multifactorial presence, and the presence of systemic facilitators such as smoking, diabetes, hypertension, endocrine disorders, mental stress, immunodeficiency, genetic factors, and nutritional deficiencies can also reduce the host’s defenses, enhance the virulence of bacterial microorganisms, and exacerbate inflammatory responses of periodontal tissues, and when the dynamic equilibrium maintained between the periodontal bacterial microorganisms and the host is dysfunctional ( dysobiosis), leading to the development of periodontal disease [26]. The present study evaluated the relationship between leisure time physical activity and serum cotinine levels with the risk of periodontitis. The study also examined the interactive effects of leisure time physical activity and serum cotinine levels on the risk of periodontitis. The results showed that low intensity of leisure time physical activity was associated with an increased risk of periodontitis, and serum cotinine levels ≥ 0.05 ng/ml were associated with an increased risk of periodontitis. There was also an interaction effect between low leisure time physical activity intensity and serum cotinine levels on periodontitis risk. These findings may inform the management of periodontitis disease burden. Crystal Marruganti et al. [27] conducted a population-based study in which 10,679 adult participants were retrieved from the 2009–2014 NHANES database to discuss the different associations of leisure-time physical activity, occupational physical activity, and periodontitis, and showed that multiple regression analysis identified high LTPA as a protective indicator of periodontitis (odds ratio [OR ] = 0.81; 95% confidence interval [CI]:0.72–0.92). The relationship between leisure-time physical activity and periodontitis may be influenced in part by biomarkers of systemic inflammation, body mass index, and comorbidities (i.e., diabetes and hypertension). Previous studies have shown an association between high levels of physical activity and periodontitis [28]. Smith L et al. [29] showed, by looking at serum inflammatory biomarkers in 3248 adults, that higher levels of leisure time physical activity were associated with lower levels of serum inflammatory biomarkers (aOR:0.60; 95% CI for highest C-reactive protein group: 0.42–0.86, aOR: 0.58; 95% CI for the highest leukocyte group: for 0.39–0.87), confirming that leisure-time physical activity may be a beneficial strategy to improve some, but not all, cardiometabolic health outcomes. Mechanisms explaining the link between leisure time physical activity and NCDs, including periodontitis, remain unclear; however, modulation of LGSI is recognized as one of the main potential pathways involved [30]. Previous studies have shown [31, 32] that higher levels of leisure time physical activity are associated with reduced levels of systemic and local (i.e., within the gingival sulcus) inflammatory biomarkers. And the possible relevance of the systemic metabolism/inflammation axis, as platelet count, white blood cells, body mass index, and diabetes partially weakened the estimate of the correlation between leisure-time physical activity and periodontitis observed in the mediation analysis. Cross-sectional studies that used a partial periodontal screening program noted that subjects with normal weight, regular physical activity, and better quality diets were 40% less likely to develop periodontal disease compared with those without these healthy lifestyles [33]. However, other studies have failed to report a significant physical activity-periodontitis relationship [34]. These conflicting results may be related to the use of different case definitions of periodontitis and the lack of a clear distinction between the quality of exposure. Previously, a Brazilian population-based study analyzing 38,539 participants aged ≥ 30 years showed that leisure-time physical activity was the only domain likely to reflect the oral health benefits of physical activity [35]. High leisure time physical activity was found to be a protective indicator of periodontitis with an OR of 0.81 [11] Our findings suggest that low intensity of leisure time physical activity is associated with a higher risk of periodontitis and these findings may provide support for our findings. A systematic review and meta-regression analysis involving 28 studies suggests that smoking adversely affects the development and progression of periodontitis [7]. Smoking is an important risk factor for many human diseases and is associated with stroke, cardiovascular disease, gastric ulcers, and oral and esophageal cancers, and smoking, as an important risk factor for the development of periodontitis, has been shown by epidemiologic results to be a high-risk factor for periodontitis, especially severe periodontitis. Many studies have shown that the risk of developing periodontitis is more than five times higher in moderate to heavy smokers than in the normal population, and it has also been shown that there is a relationship between the recurrence of periodontitis during maintenance and smoking, as well as a relationship between the severity and the number of doses. For a comparison of risk values for developing severe alveolar bone resorption, light smokers were 3.25 times more likely to be non-smokers, while heavy smokers were 7.28 times more likely to be non-smokers. This shows that smoking increases the likelihood of loss of attachment level of periodontal tissues and bone tissue resorption. Smoking may increase the risk of apical periodontitis [36], which is also supported by several systematic reviews showing that smoking is associated with an increased prevalence of periodontitis [37, 38]. A Mendelian randomized study suggests that smoking is associated with periodontitis [39]. Considering the underestimation of smoking when self-reporting smoking status [40], the cotinine test provides a quantitative and accurate reflection of the body’s short-term tobacco exposure level and is suitable for assessing smoking status and intensity [41, 42]. In our study, we observed that serum cotinine levels ≥ 0.05 ng/mL were associated with an elevated risk of periodontitis. In addition, this study found a synergistic effect between low spare time physical activity and serum cotinine levels ≥ 0.05 ng/ml, which affects the risk of periodontitis. In a study by Ciele et al. [43], extreme physical inactivity, when combined with smoking, exacerbated lung inflammation and emphysema and accelerated body and muscle weight loss. Another study found that smoking and physical inactivity may increase the risk of premature death in older hypertensive patients [44]. In the current study, the relationship between body mass index and periodontitis was also illustrated by comparing subgroups delineated by body mass index < 25 kg/m^2^ and body mass index ≥ 25 kg/m^2^ The results showed that the periodontitis group had a higher percentage of participants with a body mass index of ≥ 25 kg/m^2^ and participants with a body mass index of ≥ 25 kg/m^2^ had a serum cotinine level of ≥ 0.05 ng/ compared to the non-periodontitis group. mL and those who were less physically active in their spare time had a higher risk of periodontitis. Overweight was defined as a body mass index of 25.0 kg/m^2^ -30.0 kg/m^2^ [45]. An association between obesity and periodontitis has been published [46, 47], although causal evidence is lacking [48]. Of note, the chronic inflammatory state and oxidative stress that lead to the development of insulin resistance may be related to the association between obesity and periodontal disease [49]. A study by Lallukka et al. [50] suggests that smokers who do not exercise or exercise moderately have the highest risk of death in the working age group. The underlying mechanism may be that higher levels of leisure time physical activity have been reported to be associated with lower levels of circulating inflammation and higher levels of antioxidants [51, 52]. Cigarette smoking is associated with a disturbed inflammatory response and host response to potential periodontal pathogens, altered subgingival microbial communities, and impaired tissue healing leading to dysregulation of tissue homeostasis [53]. Dentists should recommend smoking cessation and make efforts to educate smokers about the adverse effects of smoking on periodontal health and implant treatment outcomes. The interaction between leisure-time physical activity and smoking highlights the importance of integrating improvements in behavioral factors and promoting an overall healthy lifestyle. Smoking cessation and vigorous physical activity may be necessary for smokers.

This study assessed the interaction of leisure-time physical activity and tobacco exposure on periodontitis risk using a large representative population based on the NHANES database. Sensitivity and subgroup analyses validated the robustness of the findings. There are some limitations of our study. First, this was a cross-sectional study that could only determine the association between leisure-time physical activity or tobacco exposure and periodontitis risk and could not infer causality. Second, the smoking status and secondhand smoke exposure of the participants were unknown, which may affect serum cotinine levels, and serum cotinine levels decline rapidly after exposure to smoking, although urinary cotinine levels are more stable than serum cotinine levels, but the NHANES did not see the associated variables, which may have biased the results of the study somewhat. Third, although the NHANES investigators were professionally trained, there may still be some reporting bias and respondent recall bias. More well-designed studies are needed in the future to validate the results of this study.

## Conclusion

In this study, we analyzed the relationship between leisure-time physical activity and serum cotinine levels and the risk of periodontitis, as well as the interaction between leisure-time physical activity and serum cotinine levels on the risk of periodontitis. We found that there was an interaction between intensity of leisure-time physical activity and smoking exposure on periodontitis risk. These findings may advise people to be more physically active in their leisure time and to quit smoking.

## Data Availability

The NHANES database used in this paper is publicly available, and we have followed the data usage protocol and relevant regulations for data processing and analysis. In this study, we used standard statistical methods and analysis procedures to preprocess and clean the data. The surveys were approved by the National Center for Health Statistics Research. Details of study implementation are available for online access NHANES Questionnaires, Datasets, and Related Documentation (cdc.gov); URL: https://wwwn.cdc.gov/Nchs/Hhanes in the data availability section
